# Solar-driven active and reusable immobilized fluorine-doped ZrO_2−*x*_ thin film photocatalyst for dye-contaminated water

**DOI:** 10.1039/d6ra01876a

**Published:** 2026-04-27

**Authors:** Mohamed S. Attia, Faisal K. Algethami, Mahmoud S. Abdel-Wahed, M. Obaida, Amer S. El-Kalliny

**Affiliations:** a Chemistry Department, College of Science, Imam Mohammad Ibn Saud Islamic University (IMSIU) Riyadh 11623 Saudi Arabia; b Water Pollution Research Department, National Research Centre 33 El Buhouth St., Dokki 12622 Giza Egypt; c Solid State Physics Department, Physics Research Institute, National Research Centre 33 El Buhouth St., Dokki 12622 Giza Egypt mohamed.obaida@rub.de mz.obaida@nrc.sci.eg

## Abstract

An immobilized fluorine-doped ZrO_2−*x*_ thin film photocatalyst was synthesized on glass substrates using the pulsed spray pyrolysis (PSP) technique for the first time. PSP is simple to control, capable of producing homogeneous thin films on a mass-production scale, and easy to use. Firstly, the fluorine-doped ZrO_2−*x*_ thin film was prepared at an optimum deposition temperature at 400 °C and 30 min spray time, and thoroughly characterized. Its performance was then evaluated for the photocatalytic degradation of the Remazol Red (RR) dye as a model organic water contaminant under solar light. The results revealed that the degradation kinetics of the RR dye were represented by pseudo-first-order, and the apparent rate of degradation increased fourfold, *i.e.*, from 78.7 × 10^−5^ min^−1^ for the ZrO_2_ thin film at 400 °C/30 min to 339 × 10^−5^ min^−1^ for the F-doped ZrO_2−*x*_ thin film at 400 °C/30 min. This is due to the presence of fluorine, which reduces the band gap of ZrO_2_ and exhibits substantial absorption in the solar spectrum instead of the UV range, in addition to the reduction in the rate of electron–hole pair recombination. The study found that the most important role in the RR dye degradation was played by the oxidizing species order: ^1^O_2_ ≈ O_2_˙^−^ > ˙OH. To sum up, the F-doped ZrO_2−*x*_ thin film is a promising immobilized photocatalyst for dye removal.

## Abstract 2

An immobilized fluorine-doped ZrO_2−*x*_ thin film photocatalyst was synthesized on glass substrates using the pulsed spray pyrolysis (PSP) technique for the first time and its solar-driven photocatalytic mechanism.

## Graphical abstract

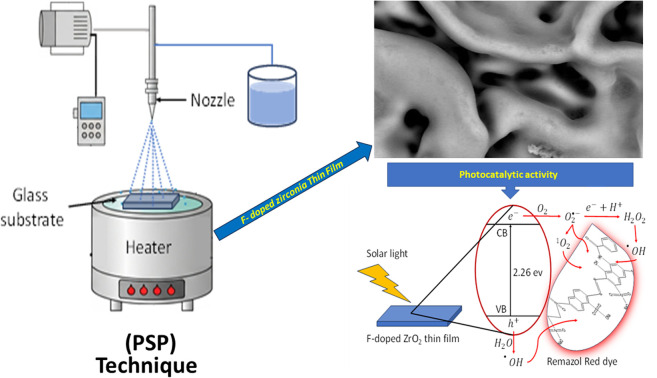

## 1 Introduction

Synthetic dyes are widely used in various sectors, and their presence in wastewater contributes to water resource contamination. These dyes are engineered to exhibit chemical and biological resistance, and their complex chemical structures render them challenging to decompose.^1^ This persistence may result in considerable environmental problems. Moreover, the aromatic chemicals generated during the breakdown of these dyes are extremely toxic and have mutagenic and carcinogenic characteristics.^2^ Azo dyes, including Remazol Red, represent a notable class of synthetic dyes that may induce genetic mutations and may result in cancer.^3^ Significant efforts have been made to eliminate these hazardous dyes from industrial effluents. However, the removal of azo dyes from wastewater remains a significant challenge.^3^

Conventional biological treatment procedures frequently do not attain substantial dye decolourization and degradation because of the high aromatic content and the stability of dye molecules.^4^ For instance, under anaerobic biodegradation circumstances, the azo bond can decompose into aromatic amines, which are carcinogenic and cannot be metabolized anaerobically, rendering them more hazardous than the original dyes.^4^ Physical approaches, such as adsorption, are non-destructive as they solely transfer dyes from the liquid phase to a solid phase, potentially resulting in secondary hazardous contamination. Consequently, employing an oxidation strategy for water treatment is very successful in this instance, especially semiconductor photocatalysis, which utilizes renewable solar energy, which can be utilized in water and wastewater treatments for the breakdown of persistent organic contaminants *via* the generation of highly oxidizing reactive oxygen species (ROS). Other advanced oxidation processes (AOPs), such as ozonation and the Fenton process, offer effective degradation but often suffer from high operational costs, strict pH requirements, or the need for continuous chemical dosing.^5,6^

Heterogeneous photo-oxidation and AOPs, utilizing solar light, are regarded as an efficient remediation technique, particularly for persistent organic water contaminants. Separating the photocatalyst from the treated effluent poses challenges, especially when in nanoparticle form, hence constraining its practical applicability.^7^ To enable the separation and recycling of the powdered photocatalyst, it can be immobilized onto a stationary support. Immobilized thin-film photocatalysts have attracted significant attention as a practical alternative to conventional powder systems for wastewater treatment applications. While suspended powder photocatalysts offer high surface area and efficient light harvesting, they suffer from major limitations including particle aggregation, difficulty in post-treatment separation, and poor recyclability, which hinder large-scale implementation.^8^ In contrast, immobilized thin films deposited on substrates such as glass or ceramics enable facile recovery and reuse without the need for filtration or centrifugation, thereby reducing operational costs and complexity.^9^ Moreover, immobilized systems exhibit enhanced mechanical stability and long-term durability, avoiding catalyst loss and aggregation during repeated cycles.^10^ These features make thin-film photocatalysts more suitable for continuous-flow and industrial-scale applications, despite their relatively lower surface area compared to powders. Consequently, from a practical point of view, the application of the photocatalyst as an immobilized thin film is superior to its powdered form regarding recovery and reuse.

Zirconia exhibits advantageous chemical and physical properties, including exceptional chemical and thermal stability, significant corrosion resistance, low thermal conductivity, high strength, and notable transparency in the near-infrared and visible spectra.^11–13^ Consequently, zirconia is used in diverse applications, including oxygen sensors, fuel cells, catalysts, catalytic supports, high-dielectric materials for large-scale integrated circuits, and gate dielectrics in metal-oxide semiconductors.^14^ A notable drawback of using zirconia as a photocatalyst is its large band gap energy (3.25–5.10 eV, depending on the preparation process).^15^ This suggests that zirconia itself cannot effectively harness sunlight, as only 2–5% of solar terrestrial radiation is ultraviolet. Moreover, the recombination of photo-generated electron–hole pairs in zirconia significantly hinders its photocatalytic efficacy in the visible light spectrum.^16^ There are many attempts to reduce the band gap and extend the lifetime of photogenerated carriers to improve the visible light photocatalytic efficiency of zirconia.

For example, the introduction of oxygen vacancies, a prevalent defect in oxides, was achieved in zirconia under extremely harsh conditions, as the energy required for oxygen vacancy formation in zirconia exceeds that of titanium oxide.^16,17^ Metallic components, including Fe, Cr, Mn, Ce, and Mg, have been utilized to adjust the band gap and enhance the photocatalytic efficacy of zirconia.^18–22^ However, nearly all metal dopants in zirconia diminish the conductive band minimum, resulting in a decreased reduction capacity of photo-generated electrons. Simultaneously, the possible toxicity and thermal instability of metal dopants raise concerns over their utilization.^16^ Significant attempts have been made to enhance the visible light sensitivity of zirconia using non-metal doping (C, N, S, *etc.*) as an alternate approach.^23–25^

Recent studies have emphasized the effectiveness of modified zirconia photocatalysts in degrading organic dyes under various irradiation conditions. For instance, Ag_0.04_ZrO_2_/rGO composites have demonstrated an 87% degradation rate of methyl orange when exposed to UV-visible light, a result that has been attributed to the reduction of the bandgap through the incorporation of reduced graphene oxide.^26^ Nitrogen doping has also been shown to be beneficial: N-doped ZrO_2_ has demonstrated efficiencies of 67% and 96% for amaranth and methylene blue (MB) under visible and UVA light, respectively.^27^ Furthermore, N-doped ZrO_2_ thin films have achieved degradation rates of over 90% for MB and rhodamine B under visible light, with band gaps reduced to 2.54–2.61 eV.^28^ Similarly, F-doped ZrO_2_ powder has achieved an impressive 89.7% degradation of methyl orange within 40 minutes under simulated solar light.^29^

Furthermore, F-doped ZrO_2−*x*_ nanotubes exhibiting visible light photocatalytic activity were prepared using a combination of anodic oxidation and subsequent annealing in an Ar atmosphere, demonstrating 83% degradation of Rhodamine B dye after 2 hours.^16^ Although zirconium oxide has been widely investigated as a photocatalyst in powder form,^30,31^ studies on immobilized ZrO_2_ thin films remain comparatively limited, particularly those fabricated using cost-effective and scalable techniques. In those systems, the photocatalytic performance was primarily governed by surface hydroxylation and defect chemistry, but visible-light response remained limited due to the intrinsically large bandgap of zirconia. Additionally, most immobilized zirconium oxide systems reported in the literature rely on conventional preparation routes such as sol–gel or sputtering, with limited emphasis on scalable spray-based thin-film fabrication and band structure engineering.

Moreover, while elemental doping has been explored to enhance photocatalytic performance, systematic investigations of fluorine-doped ZrO_2−*x*_ thin films in immobilized configurations are still scarce. The novelty lies in combining (i) thin-film immobilization on glass substrates for practical recovery and reuse, (ii) fluorine-induced bandgap engineering through oxygen-vacancy formation and impurity-state modulation, and (iii) a single-step deposition route compatible with large-area fabrication. This integrated approach differentiates the present study from earlier immobilized ZrO_2_ systems that rely primarily on intrinsic UV activity (wide bandgap energies (typically 3.2–5.0 eV, limiting their activity predominantly to the UV region) without deliberate electronic structure modification.^32^

Fluorine-doped zirconia thin films have been fabricated using different techniques reported in the literature, including atomic layer deposition,^33^ RF magnetron sputtering,^34^ low-temperature combustion,^35^ and molecular beam epitaxy.^36^ The potential enhancement of zirconia with fluorine prompts us to produce F-doped films, which have not yet been synthesized for photocatalytic activity. This study presents the preparation of an immobilized F-doped ZrO_2−*x*_ thin film photocatalyst deposited on glass substrates, which is cost-effective, easy to clean, and more manageable than alternative substrates, by using the pulsed spray pyrolysis technique. This system is simple, easily controlled, and capable of producing homogeneous thin films on a mass-production scale.^37^ The synthesized solar active thin films were evaluated, and their efficacy and reusability for the degradation of RR dye were examined.

## 2 Experimental methods

### 2.1. Preparation of F-doped ZrO_2−*x*_ thin films

**Fig. 1:**
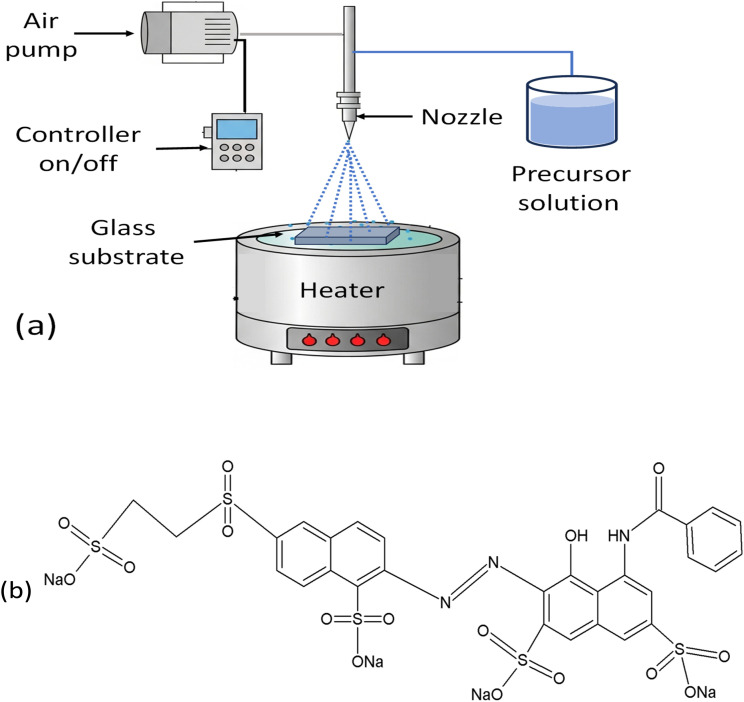
(a) Schematic diagram for pulsed spray pyrolysis system. (b) Chemical structure of Remazol Red F3B dye.

To overcome limitations such as poor repeatability, inconsistent spray deposition, and non-uniform film homogeneity in previous systems. A modified pulsed (intermittent) spray pyrolysis setup (Fig. 1a) was developed and assembled for the synthesis of zirconium dioxide and F-doped ZrO_2−*x*_ thin films using zirconium oxychloride (ZrOCl_2_·8H_2_O, Merck 99%) and ammonium fluoride (NH_4_F, 99%). Both ZrO_2_ and F-doped thin films were deposited onto cost-effective soda-lime glass substrates following standard cleaning procedures. Pure and doped films were deposited under the following optimized conditions: an aqueous precursor solution containing 0.05 M of ZrOCl_2_ (which is lies within the commonly reported range for spray pyrolysis deposition of uniform oxide thin films^38,39^),and 0.2 M (to compensate for possible fluorine loss during deposition, an excess amount of F precursor was deliberately introduced as reported previously in literature^40–45^) of NH_4_F was dissolved in (100 mL) distilled water and stirred until a homogeneous, clear solution was obtained; the nozzle-to-substrate distance was kept at 30 cm, and the carrier gas flow rate of 25 L h^−1^ (filtered air) was used. Pure and doped samples were deposited at different spray times of 15, 30, and 45 minutes, with an optimum deposition temperature at 400 °C and 30 min with 1 s ON and 4 s OFF of the spraying pulses. At this temperature, the precursor decomposes efficiently without causing thermal stress or film cracking and exhibits good wetting between the film and substrate, issues that were occasionally observed at higher temperatures. Additionally, keeping the spray time at 30 minutes helps control film thickness, which is lower for short spray times, and prevents excessive particle agglomeration, which has been observed with longer spray times.

### 2.2. Characterization of the prepared thin films

The structural analysis of the pure and F-doped ZrO_2−*x*_ films was carried out by X-ray diffraction using XRD (Empyrean, PANalytical, Netherlands) to determine and identify the crystallographic phases of the prepared samples over the range 10° ≤ 2*θ* ≤ 80°. The surface morphology of the deposited films was examined using a field emission scanning electron microscope (FESEM, Quanta FEG 250 equipped with a field emission gun, FEI Company, Netherlands). Elemental microanalysis and the spatial distribution of elements (mapping) of the prepared samples were performed using energy-dispersive X-ray spectroscopy (EDX) integrated with the FESEM system. The measurement of optical absorption of the deposited films was conducted using the Jasco V630 spectrophotometer in the UV/VIS regions. The determination of fluorescence lifetime was achieved through the utilization of an FS5 spectrofluorometer (Edinburgh, UK) that was employed to excite the sample at a specific wavelength of 350 nm.

### 2.3. Degradation of RR by the immobilized photocatalysts

The photocatalytic activities of the synthesized films were evaluated by degrading Remazol Red (RR), a model dye contaminant in water, as shown in Fig. 1b. To do this, a 2.5 × 2.5 cm^2^ film was initially affixed to a 2 cm-high holder within a 100 mL beaker using silicone adhesive. Subsequently, 60 mL of a 10 mg L^−1^ RR solution at its natural pH was introduced into the beaker, which was placed in the dark for 60 min and agitated using a magnetic stirrer to reach adsorption–desorption equilibrium. The beaker was vertically irradiated in a solar system (UVA CUBE 400, Dr Hönle AG UV Technology, Germany) using a halogen high-pressure lamp (model: SOL 500) with a wavelength range from 295 to 780 nm. The bulb of this device provides radiation that simulates natural sunshine (1000 W m^−2^). At specific time intervals, online measurement of RR was quantified by measuring absorbance at 542 nm using a UV-visible spectrophotometer (JASCO V630, Japan). The following equation (eqn (1)) is employed to represent the non-linear regression for pseudo-first-order:

1 *C* = *C*_0_*e*^−*kt*^,

where, *C*_0_ and *C* in mg L^−1^ are the concentrations of the RR dye at *t*_0_ (0 min) and *t* (min), respectively, and *k* (min^−1^) is the rate constant of pseudo-first-order. The reusability of the F-doped ZrO_2−*x*_ thin film photocatalyst was also evaluated. To do this, after the second run, the immobilized photocatalyst was washed and irradiated in distilled water before being reused in the subsequent cycle of dye degradation to remove any adhering byproducts and allow self-purification. The caption of the figures includes all the experimental conditions for the degradation.

### 2.4. Trapping of reactive species

The scavengers are employed to examine the function of reactive species produced during the photocatalytic process as described elsewhere.^46^ Ammonium oxalate (AO), *para*-benzoquinone (*p*-BQ), sodium azide (SA), and isopropyl alcohol (IPA) can trap the holes (h^+^), superoxide radicals (O_2_˙^−^), singlet oxygen (^1^O_2_), and the hydroxyl radicals (˙OH), respectively. Each quencher at a concentration of 100 mM was individually introduced to the RR solution during the photocatalytic experiment, utilizing the F-doped ZrO_2−*x*_ thin film synthesized at 400 °C with 30 min spraying duration.

## 3 Results and discussion

### 3.1. Characterization of the prepared thin films

**Fig. 2:**
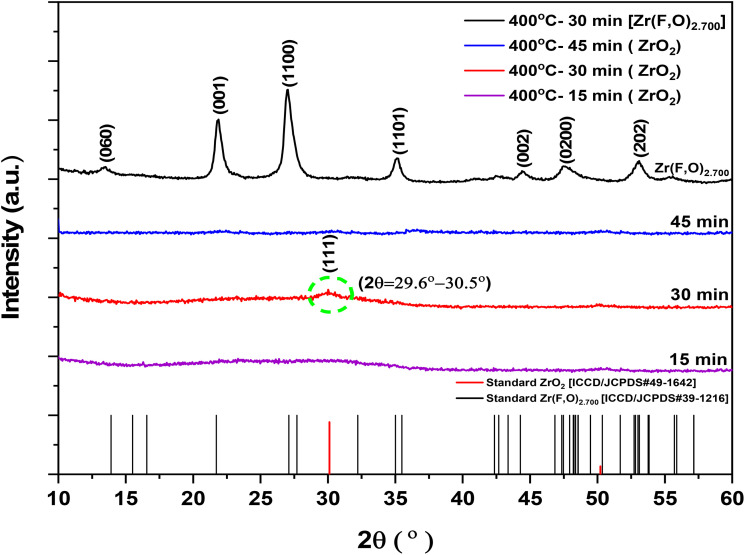
XRD patterns of pure-ZrO_2_ (15, 30, and 45 min) and F- doped ZrO_2−*x*_ (30 min) thin films deposited at a substrate temperature of 400 °C.

Fig. 2 presents the X-ray diffraction (XRD) analysis of pure and F-doped ZrO_2−*x*_ thin films deposited at a hotplate temperature of 400 °C with spray times of 15, 30, and 45 minutes. As shown in Fig. 2, the XRD pattern of the ZrO_2_ films deposited at a shorter spray time of 15 min exhibits a completely amorphous structure, while samples prepared at a spray time of 45 min exhibit an amorphous nature similar to those observed for the 15 min deposition time, which is ascribed to the long spray time;^38^ therefore, the film deposited for 30 min represents the optimal deposition condition. This is evidenced by the appearance of a broad hump peak of an amorphous structure in the 2*θ* range of 29.6° −30.5°, corresponding to the (111—highlighted by the dashed green circle in Fig. 2) compared with standard (ICDD/JCPDS #49-1642) reference data sheets reflection, which makes it difficult to clearly distinguish between the tetragonal and cubic zirconium oxide phases due to their overlapping peak positions.^47,48^

This overlap, likely caused by internal film stress, suggests the presence of a poorly crystallized monoclinic phase or a nearly amorphous structure, which hinders proper crystal growth.^49^ In contrast, the black curve for F-doped ZrO_2−*x*_ films exhibits sharp, well-defined peaks, indicating a highly crystalline phase that improves with the F-incorporation with a crystalline structure of zirconium oxide fluoride [Zr(F, O)_2.700_], where the number [2.700] reflects a mixed-anion site occupancy, suggesting a substitution ratio of O^2−^ for F^−^ in the lattice. The recorded diffraction peaks match well with the 2 theta angles at 13.4°, 21.7°, 27°, 35.2°, 44.4°, 47.5°, and 53.09° of (060), (001), (1100), (1101), (002), (0200) and (202) planes, respectively, in good agreement with the standard XRD reference (ICDD/JCPDS #39-1216).

The absence of the ZrO_2_ peak suggests that both ZrO_2_ crystallization suppression and complete phase transformation occur due to fluorine incorporation. Using the Scherrer formula (eqn (2)).^50^

2 *C* = *Kλ*/*β* cos(*θ*),

where the Scherrer constant *k* = 0.94, *λ* is the wavelength of CuK_α_ radiation of 1.5408 Å, and *β* is the full width at half maximum (FWHM). The average crystallite size for ZrO_2−*x*_ F-doped samples was calculated to be approximately ≈10 nm. Although the spray time (*i.e.*, the thickness-related parameter) was varied, XRD analysis indicates poorly crystalline at 15 and 45 min. Therefore, thickness measurements were not explicitly conducted, as the study primarily focuses on the structural modifications induced by fluorine incorporation rather than thickness-dependent effects.

### 3.2. FESEM and EDX morphological analysis

**Fig. 3:**
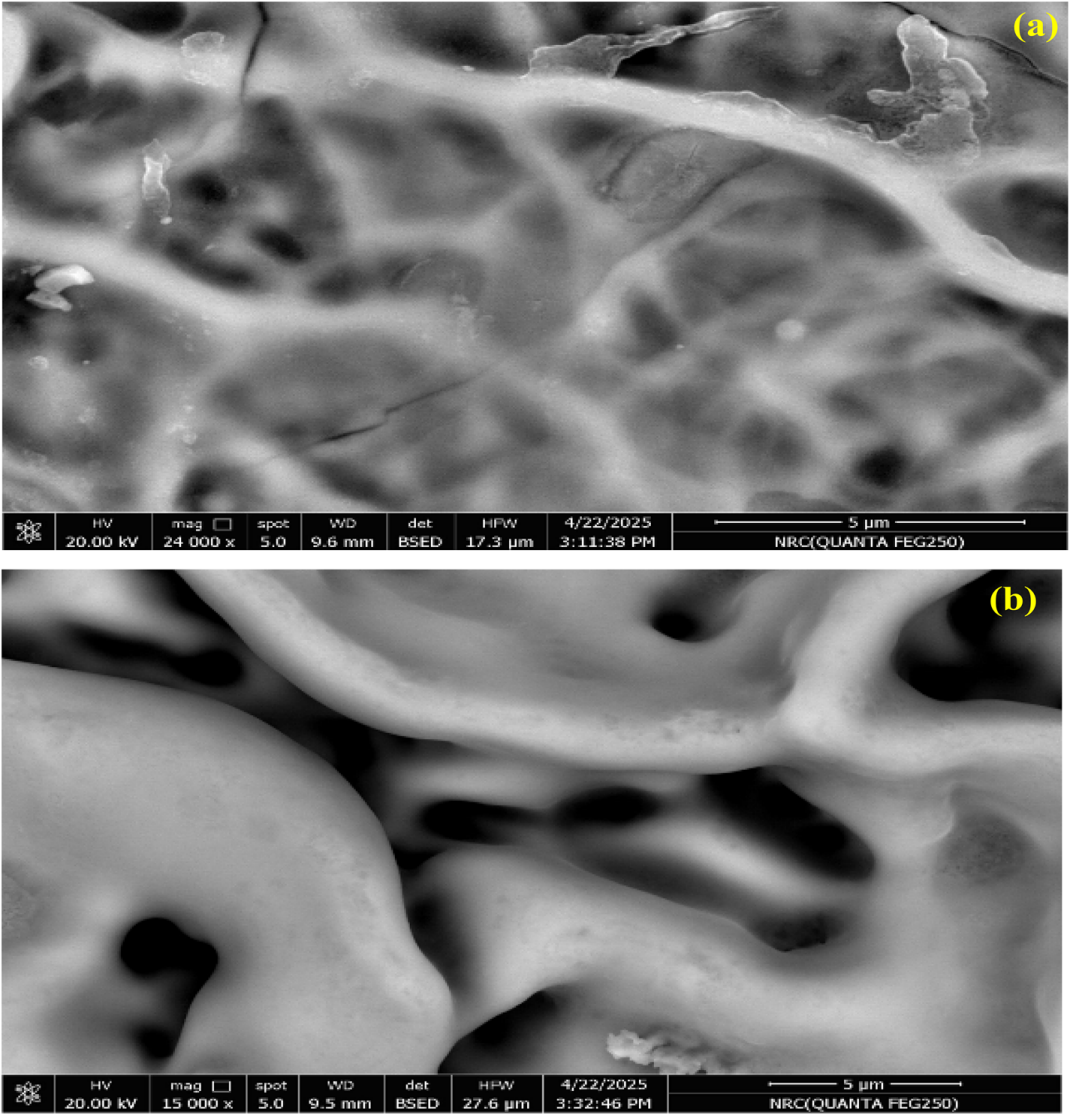
FESEM images of (a) ZrO_2_ and (b) F-doped ZrO_2−*x*_ thin films. Pure ZrO_2_ shows a fine-grained surface network structure, while the doped films exhibit a denser, smoother morphology due to fluorine incorporation.

**Fig. 4:**
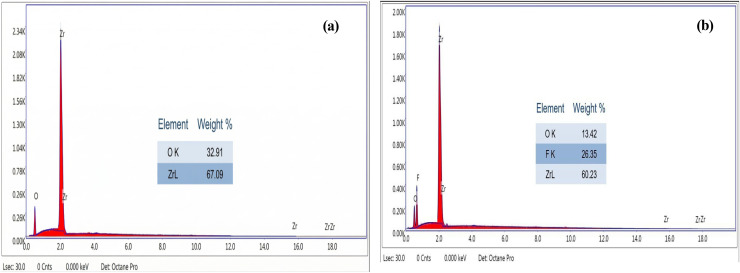
EDX measured spectra of (a) pure ZrO_2_, and (b) F-doped ZrO_2−*x*_ deposited films.

**Fig. 5:**
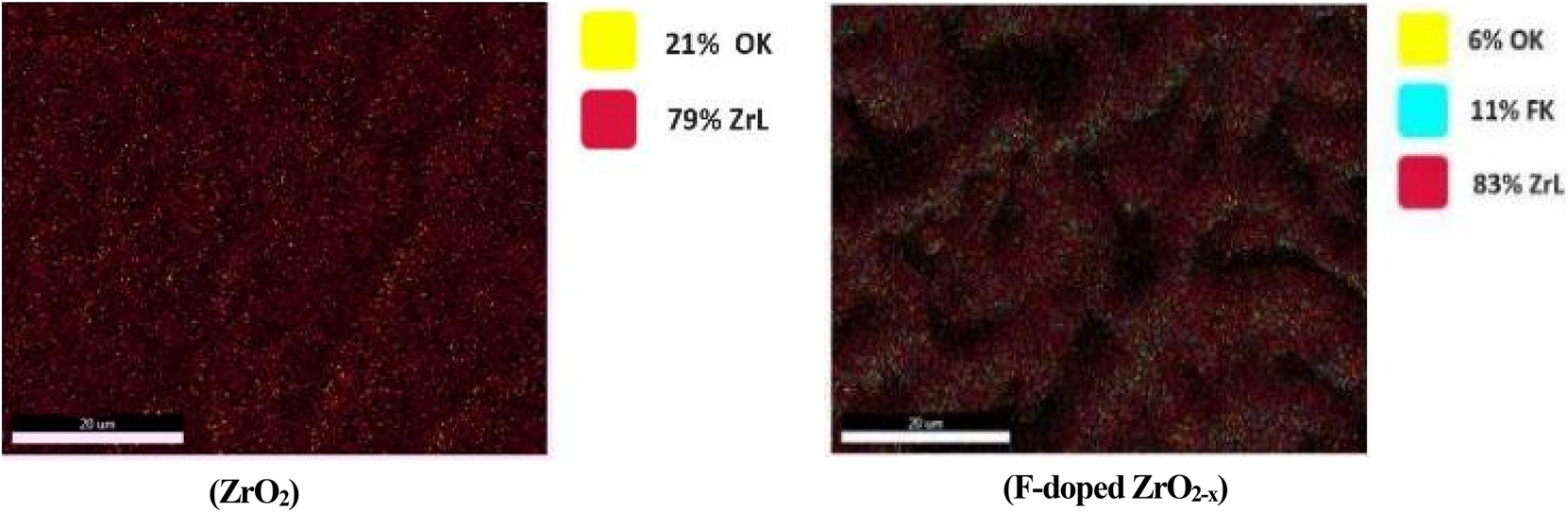
EDX elemental mapping of ZrO_2_ and F-doped ZrO_2−*x*_ phases.

The FESEM images in Fig. 3(a and b) reveal distinct microstructural differences between the two films, in agreement with the XRD results. In Fig. 3a, the ZrO_2_ film displays a highly cracked, fine-grained surface network characteristic of an amorphous matrix, consistent with the absence of sharp diffraction peaks and the presence of only a weak, broad feature in the XRD pattern. In contrast, in Fig. 3b, the F-doped ZrO_2−*x*_ film exhibits a coarser, interconnected grain-like structural morphology with smoother and more consolidated features, reflecting the well-defined crystalline nature of [Zr(F, O)_2.700_] confirmed by its sharp XRD reflections. These observations collectively demonstrate that fluorination at 400 °C promotes the transition from an amorphous oxide to a fully crystallized fluoride phase with improved structural ordering. Energy-dispersive X-ray spectroscopy was used to analyze the elemental composition of two zirconium-based phases: pristine zirconium dioxide and zirconium oxide fluoride. The EDX spectrum of ZrO_2_ in Fig. 4a shows distinct peaks for zirconium (Zr) and oxygen (O), with no additional elements detected, confirming its purity. In contrast, the F-doped ZrO_2−*x*_ spectrum in Fig. 4b exhibits additional signals for fluorine (F), with an obtained ratio of Zr : F : O of (60.23 : 26.35 : 13.42), which confirms the presence of F atoms in the zirconium oxide matrix, indicating successful halide incorporation. The consistently high Zr peak intensities in both spectra affirm zirconium as the dominant element. These findings highlight the compositional distinction between the phases and confirm fluorine integration into the zirconia matrix in the form of the zirconium oxide fluoride phase. Energy-dispersive X-ray spectroscopy mapping was conducted to evaluate the elemental composition and spatial distribution in the ZrO_2_ and F-doped ZrO_2−*x*_ films, as shown in Fig. 5. The mapping analysis consistently confirmed zirconium (Zr) and oxygen (O) as the principal elements across all investigated regions. In the recorded (left-hand-sided) image, zirconium (Zr) is the dominant element, accounting for 79% (ZrL), with oxygen (OK) present at 21%, indicating a strong oxide phase and confirming the formation of the zirconium dioxide phase. The elemental dispersion appears uniform, consistent with a well-formed zirconia matrix, and the absence of significant elemental impurities supports the high purity and structural integrity of the zirconium oxide phase. In contrast, the ZrO_2−*x*_ F-doped phase (right-hand-sided image) shows a more complex composition with 83% ZrL, reduced oxygen content (6% OK), and the introduction of fluorine (11% FK), which is in good agreement with the obtained structural XRD measurements. This elemental shift suggests partial substitution of oxygen by fluorine in the zirconia structure, characteristic of the formation of the Zr(F, O)_2.700_ phase. The fluorine distribution appears uniformly integrated with Zr, indicating successful doping or formation of a fluorinated zirconia matrix.

### 3.3. Optical measurements and analysis

**Fig. 6:**
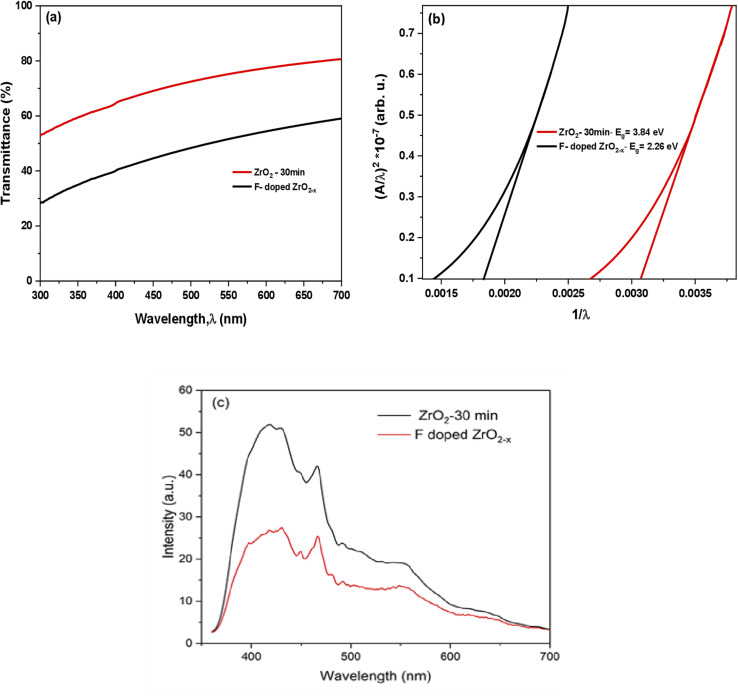
(a) Optical transmittance spectra of pure ZrO_2_ and F-doped ZrO_2−*x*_ films deposited at 400 °C for a spray time of 30 min, (b) corresponding optical bandgap plots determined using the ASF method from UV-vis measurements, and (c) photoluminescence spectra.

The optical transmission spectrum of the well-deposited pure and F-doped ZrO_2−*x*_ thin film was recorded in the wavelength range of 300–700 nm using a UV-vis spectrophotometer. As shown in Fig. 6a, the films exhibit relatively high optical transparency in the visible region, with transmittance gradually increasing toward longer wavelengths, with transmittance increasing gradually with wavelength, yielding an average transmittance of 80% for the sample deposited at a spray time of 30 minutes. The slightly reduced transparency observed for the F-doped sample is attributed to defect-related absorption associated with fluorine incorporation and to oxygen-vacancy-induced localized states, which introduce additional light-scattering and absorption centres within the film. The optical bandgap energies of the pure and doped films were determined from the absorption spectrum without any additional information, such as film thickness or reflectance spectra, using extrapolation of Tauc's model and the absorption spectrum fitting (ASF) method, as reported in ref. 50 and 51. The optical bandgap values were estimated from the extrapolated lines with the *X*-axis of the plot of (*A*/*λ*)^2^*versus* 1/*λ* using the (ASF) model, assuming allowed direct electronic transitions, as shown in Fig. 6b.

The optical bandgaps of the pure and F-doped ZrO_2−*x*_ thin films were determined to be 3.84 eV and 2.26 eV, respectively, with the bandgap of the pure film being consistent with the reported value for zirconia in literature.^15^ The narrowing bandgap of the F-doped ZrO_2−*x*_ is ascribed to the creation of oxygen vacancies and fluorine-associated localized impurity states adjacent to the conduction band, thereby validating the effective integration of F atoms into the zirconium oxide matrix, consistent with similar decreases in bandgap energy documented for F-doped zirconium oxide in the literature.^16,29^ This bandgap reduction enhances visible-light absorption, underscoring the potential of fluorine doping to improve the optical efficacy of ZrO_2_-based films for photocatalytic applications.

The photoluminescence spectra of the synthesized films are illustrated in Fig. 6c. Pure ZrO_2_ films, deposited at 400 °C for 30 minutes, exhibit broad peaks over the wavelength range 350–700 nm. These peaks can be attributed to the emission from defect or trap levels. Conversely, depositing F-doped films at the same temperature and duration resulted in a significant decrease in the intensity of the PL peaks associated with F-doped ZrO_2−*x*_. This observation indicates the influence of fluorine in reducing the recombination rate of excited charge carriers in the doped sample, thereby suggesting an enhanced photo-oxidation efficiency for this particular sample.

### 3.4. Photocatalytic activity of the prepared thin films

**Fig. 7:**
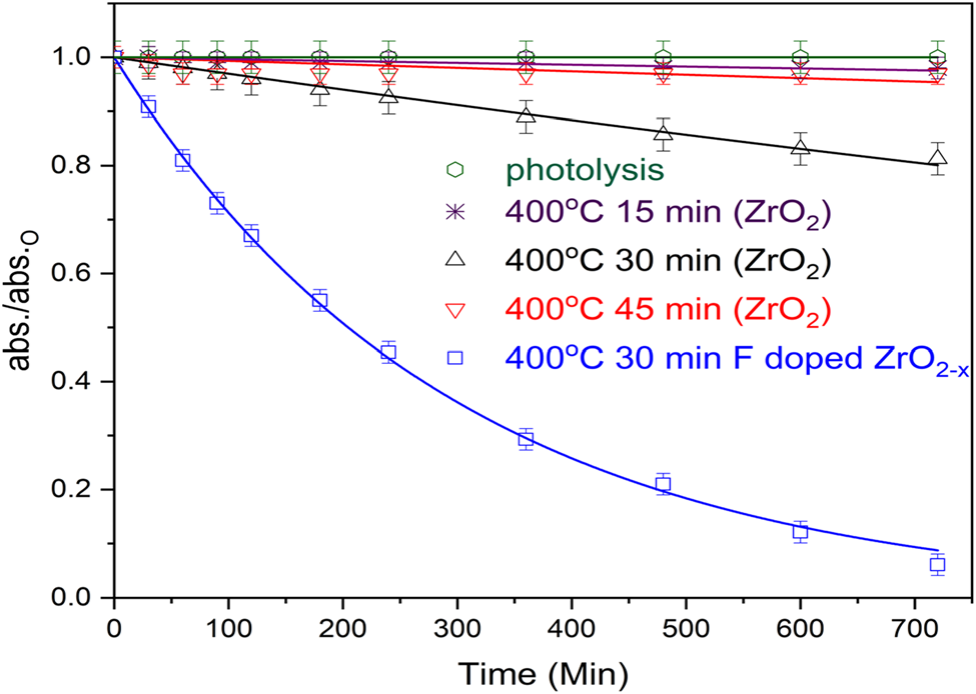
The photocatalytic degradation profile of ZrO_2_ deposited at 400 °C for (15, 30, and 45 min) and F-doped ZrO_2−*x*_ (400 °C/30 min) thin films prepared, RR = 10 mg L^−1^ at natural pH.

The photocatalytic efficacy of the prepared thin films (2.5 × 2.5 cm^2^) was evaluated for the degradation of RR, a model dye contaminant. Before initiating the irradiation process, the adsorption experiment was conducted in the dark to ensure equilibrium adsorption. This phenomenon is illustrated in Fig. S1. As demonstrated in Fig. 7, the relationship between RR concentration and irradiation time is illustrated, utilizing ZrO_2_ films deposited at 400 °C for durations of 15, 30, and 45 minutes, in conjunction with F-doped (400 °C/30 minutes) layers, employing nonlinear fitting of first-order reaction kinetics as the *R*^2^ values were close to unity.

The apparent photodegradation rate constants are presented in Table 1, RR exhibited negligible photolysis under solar light. Furthermore, as demonstrated in the XRD section (*cf.*Fig. 2), the photodegradation rate of ZrO_2_ at 15 and 45 minutes is marginally enhanced due to its amorphous structure. Conversely, during the 30-minutes deposition phase, the photodegradation rate of RR increases due to the development of zirconia crystals. These crystals can absorb light and generate electron–hole pairs, thereby facilitating the photodegradation of the dye. In contrast, the F-doped ZrO_2−*x*_ (400 °C/30 min) thin film sample demonstrated the most rapid photodegradation rate. This phenomenon can be attributed to the presence of fluorine, which has been demonstrated to reduce the band gap of ZrO_2_ and exhibit substantial absorption in both the ultraviolet (UV) and visible spectrums. Furthermore, fluorine has been demonstrated to reduce the rate of electron–hole pair recombination as a consequence of these effects.

**Table 1:** The apparent first-order rate constants in terms of irradiation time obtained for the degradation experiments of different thin films

Experiment	*K*_app_ (min^−1^)	*R* ^2^
Photolysis	3.5 × 10^−5^	0.6
ZrO_2_ thin film at 400 °C/15 min	30.8 × 10^−5^	0.99
ZrO_2_ thin film at 400 °C/30 min	78.7 × 10^−5^	0.99
ZrO_2_ thin film at 400 °C/45 min	47.3 × 10^−5^	0.99
F-doped ZrO_2−*x*_ thin film at 400 °C/30 min	339 × 10^−5^	0.99

### 3.5. The suggested F-doped ZrO_2−*x*_ immobilized thin film photocatalyst action mechanism

**Fig. 8:**
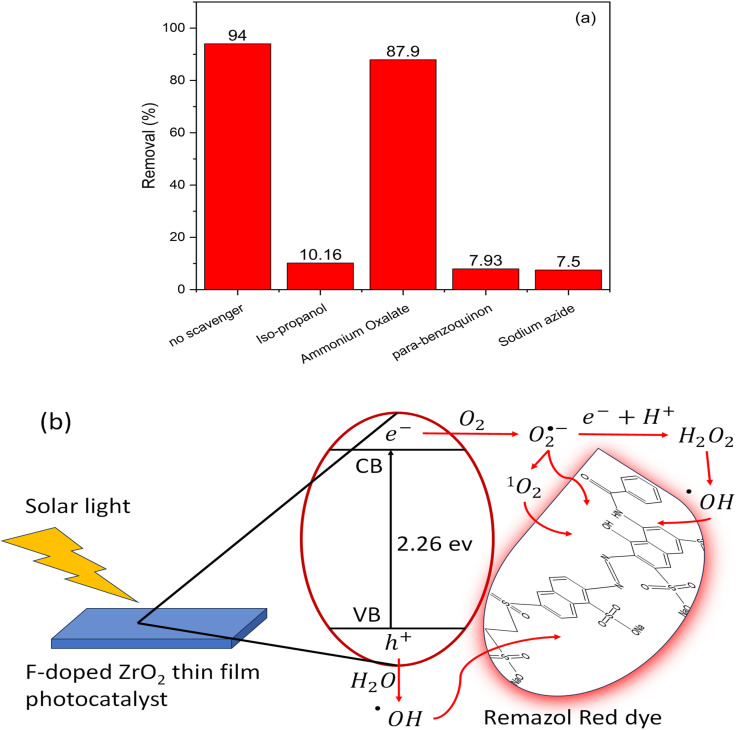
(a) Scavengers' effect on RR removal efficiency of the F-doped ZrO_2−*x*_ thin film prepared at 400 °C with a spraying time of 30 min in the presence of different scavengers (b) Suggested photocatalytic mechanism for RR degradation by the doped immobilized thin film photocatalyst.

The role of the reactive species generated in the photocatalytic process was investigated by using scavengers for the reactive oxidative species. AO, *p*-BQ, SA, and IPA can trap h^+^, O_2_˙^−^, ^1^O_2_, and ˙OH, respectively.^46^ RR degradation using F-doped ZrO_2−*x*_ immobilized thin film prepared at 400 °C with a spraying time of 30 min in the presence of scavengers is shown in Fig. 8a. The % removal of Remazol Red decreased after adding *p*-BQ, SA, and IPA, indicating that O_2_˙^−^, ^1^O_2_, and ˙OH, respectively, have a major role in the RR degradation with very little effect for h^+^. The contribution of oxidizing species in the dye degradation process follows the order: ^1^O_2_ ≈ O_2_˙^−^ > ˙OH. Based on the findings of RR degradation and generated carrier's scavenger experiments, a mechanism explaining the degradation of Remazol Red utilizing F-doped ZrO_2−*x*_ immobilized thin film in the presence of solar light was proposed (Fig. 8a). Fig. 8b shows a schematic diagram of the proposed photocatalytic mechanism for F-doped ZrO_2−*x*_ thin film. The solar light can activate the doped samples, and the excited charge carrier transfer process is shown in Fig. 8b, where the produced electrons are transferred from the valence band (VB) to the conduction band (CB).

˙OH radicals are produced when the generated h^+^ reacts with the adsorbed H_2_O and OH^−^ on the photocatalyst's surface. Conversely, e^−^ transfer from CB to the surface of the catalyst and F atoms lowers the rate of e^−^/h^+^ recombination and increases the photocatalytic activity. Superoxide radicals (O_2_˙^−^) are created when e^−^ reacts with the dissolved oxygen in the water.^16^ The following equations provide a more comprehensive explanation of the mechanism:

3 F–ZrO_2−*x*_ + *hν* → F–ZrO_2−*x*_(h^+^, e^−^)

4 F–ZrO_2−*x*_(h^+^, e^−^) + H_2_O + OH^−^ + O_2_^1^O_2_ + O_2_˙^−^ + ˙OH

5 ^1^O_2_ + O_2_˙^−^+ ˙OH + remazol red photodegradation product

**Fig. 9:**
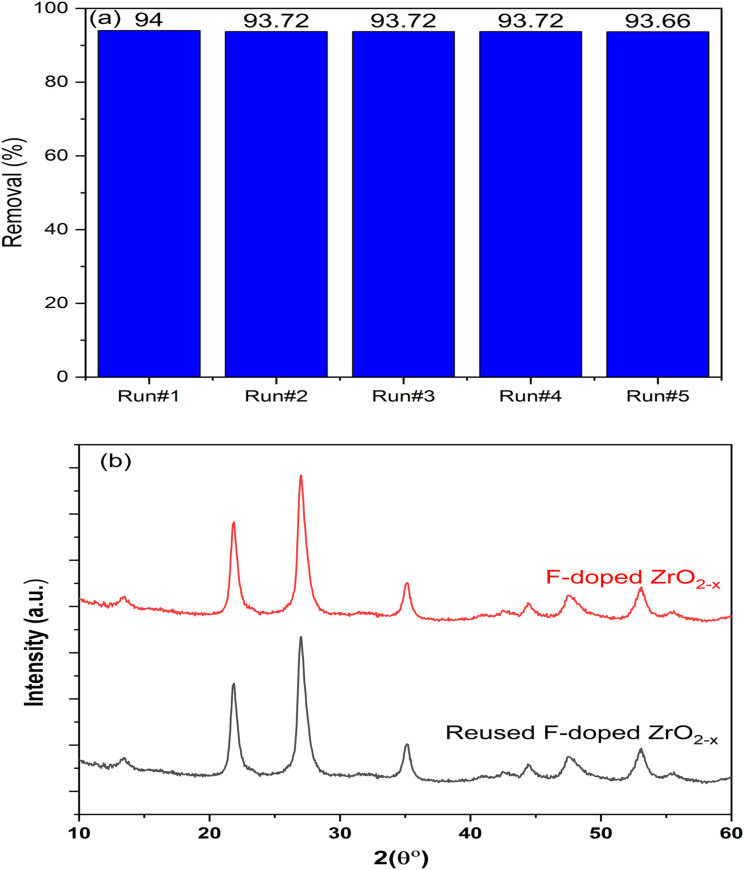
(a) Reusability of the F-doped ZrO_2−*x*_ thin film prepared at 400 °C with a spraying time of 30 min, *C*_0_ of RR is 10 mg L^−1^ at natural pH, and (b) the XRD patterns of the photocatalyst have been measured at multiple points: before and after five successive runs.

These radicals then combine with h^+^ to form singlet oxygen (^1^O_2_) species. Moreover, O_2_˙^−^ reacts with e^−^ and H^+^ to produce hydrogen peroxide (H_2_O_2_), which, when exposed to solar light, can produce ˙OH. Conversely, the F-doped ZrO_2−*x*_ thin film reusability process is a significant concern, as it enhances the economic efficiency of the treatment process. As illustrated in Fig. 9a, a five-cycle reusability test was conducted for the F-doped thin film that was prepared at 400 °C with a spraying time of 30 minutes. The RR photodegradation test was used to assess the material's stability under various conditions. After the initial cycle, a slight decrease in removal efficiency was observed when the photocatalyst was used without washing and irradiation in the presence of distilled water. Subsequent cycles revealed no alteration in RR removal efficiency, as self-purification was enabled by irradiating the photocatalyst before each run. The F-doped ZrO_2−*x*_ sample that was utilized was gathered following five cycles and analyzed through XRD (refer to Fig. 9b) to observe changes in its crystallographic structure. It was demonstrated that the XRD pattern of the employed photocatalyst exhibited a high degree of similarity with that of the freshly prepared sample. The findings of this research support the hypothesis that the F-doped ZrO_2−*x*_ thin film serves as an effective and stable photocatalyst for a long time, suitable for extended practical use.

To contextualize the enhanced photocatalytic performance of the optimized F-doped ZrO_2−*x*_ thin film, we compared it with previously reported ZrO_2_-based photocatalysts (Table 2). Several studies demonstrate that dopant introduction (*e.g.*, N, Ag, F) or formation of heterostructures (*e.g.*, rGO composites) significantly narrows the optical bandgap of zirconium oxide and extends absorption into the visible region, resulting in improved degradation of organic dyes under UV-vis or solar irradiation.

**Table 2:** Comparison of the photocatalytic performance of ZrO_2_-based photocatalysts for organic dye degradation with the present study

Photocatalyst	Dye used	Light source	Bandgap (eV)	*K*_app_ (min^−1^)	Degradation efficiency (%)	Ref.
Ag_0.04_ZrO_2_/rGO	Methyl orange (10 mg L^−1^)	UV-vis (100 min)	−(Reduced *via* rGO)	2.12 × 10^−2^	87%	26
N-doped ZrO_2_	Amaranth/MB (10 mg L^−1^)	Visible & UVA	∼2.6–3.6	N/A	67% (AM), 96% (MB)	27
F-doped ZrO_2_ powder	Methyl orange (MO) (10 mg L^−1^)	Simulated solar	Reduced *vs.* pure ZrO_2_	63 × 10^−3^	89.7% (40 min)	29
N-doped ZrO_2_ thin film	MB/RhB (5 mg L^−1^)	Visible	2.54–2.61	2.549 × 10^−2^	Up to ∼90 + %	28
Present F-doped ZrO_2−*x*_ thin film	Remazol red (RR) (10 mg L^−1^)	Visible	∼2.26	339 × 10^−5^	∼94%	This work

These results align with our findings and reinforce the role of dopant-induced bandgap modification in enhancing the visible-light photocatalytic activity relative to undoped zirconium oxide materials.

## 4 Conclusions

The F-doped ZrO_2−*x*_ thin film photocatalyst on glass substrates was successfully prepared for the first time by the PSP technique and evaluated. A homogeneous and stable thin film was obtained with optimized deposition conditions (400 °C, 30 min spray time). This thin film exhibits markedly enhanced photocatalytic activity under solar irradiation. The incorporation of fluorine results in a fourfold increase in the degradation rate of Remazol Red dye compared to undoped ZrO_2_ thin films. This is due to the action of fluorine, which significantly reduced the band gap, improved solar absorption, and suppressed electron–hole recombination. The experiments with oxidizing species scavengers revealed that singlet oxygen and superoxide radicals were the dominant species for the degradation of RR dye, with hydroxyl radicals playing a secondary role. These findings highlight the scalability and simplicity of the PSP technique alongside the superior performance of F-doped ZrO_2−*x*_ film solar photocatalyst, positioning this material as a promising candidate for practical, heterogeneous immobilized photocatalytic systems for the removal of organic water contaminants such as dyes.

## Ethics approval

On behalf of all authors, the corresponding author states that there are no ethical issues and the research does not involve studies on humans or their data.

## Author contributions

Mohamed S. Attia: conceptualization, experimental management, formal analysis, investigation, and writing—review and editing. Faisal K. Algethami: investigation, visualization, validation, review, and editing. Mahmoud S. Abdel-Wahed: investigation, experimental management, methodology, visualization, writing—original draft. M. Obaida (Corresponding Author): conceptualization, investigation, data curation, experimental management, methodology, validation, resources, writing—original draft, writing—review & editing. Amer S. El-Kalliny: conceptualization, formal analysis, investigation, and review and editing.

## Conflicts of interest

The authors state that there is no conflicts of interest.

## Supplementary Material

RA-016-D6RA01876A-s001

## Data Availability

The authors confirm that the data supporting this article have been included in the main text and the supplementary information (SI). Supplementary information: the adsorption profile of ZrO_2_ deposited at 400 °C for (15, 30, and 45 min) and F-doped ZrO_2−*x*_ (400 °C/30 min) thin films prepared, RR = 10 mg L^−1^ at natural pH. See DOI: https://doi.org/10.1039/d6ra01876a.
