# Supplementary material for: Solar-driven active and reusable immobilized fluorine-doped ZrO2−x thin film photocatalyst for dye-contaminated water
Source: RSC Adv. 2026 Apr 27;16(24):21805–16. doi: 10.1039/d6ra01876a (PMC13111980; doi:10.1039/d6ra01876a)
Supplement: RA-016-D6RA01876A-s001 [file RA-016-D6RA01876A-s001.pdf]

Supplementary Information (SI) for RSC Advances.
This journal is © The Royal Society of Chemistry 2026

Supplementary Information

Solar-driven active and reusable immobilized fluorine-doped ZrO_{2-x} thin film photocatalyst for dye-contaminated water

Mohamed S. Attia[1], Faisal K. Algethami[1], *Mahmoud S. Abdel-Wahed*[2], *M. Obaida*[3*], *Amer S. El-Kalliny*[2]

[1]*Chemistry Department, College of Science, Imam Mohammad Ibn Saud Islamic University (IMSIU), Riyadh 11623, Saudi Arabia*
[2]*Water Pollution Research Department, National Research Centre, 33 El Buhouth St., Dokki, 12622 Giza, Egypt*

[3]*Solid State Physics Department, Physics Research Institute, National Research Centre, 33 El Buhouth St., Dokki, 12622 Giza, Egypt*

[*] **Corresponding Author:** Mohamed Obaida; email: mohamed.obaida@rub.de ; mz.obaida@nrc.sci.eg

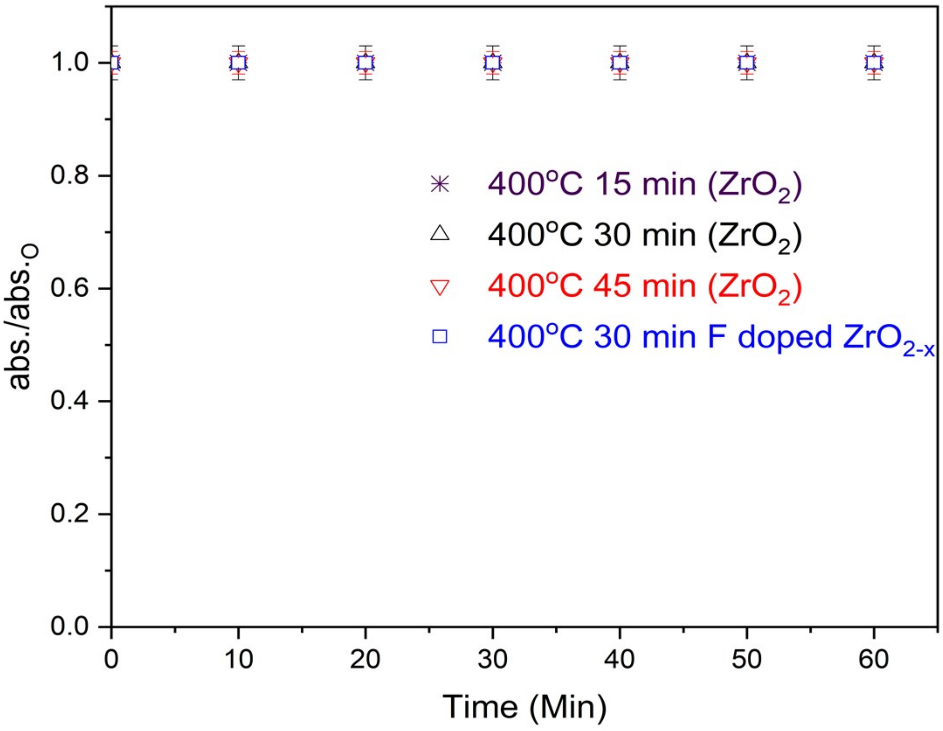

Fig. S1 The adsorption profile of ZrO_2 deposited at 400°C for (15, 30, and 45 min) and F-doped ZrO_{2-x} (400°C/30 min) thin films prepared, RR =10 mg/L at natural pH.
